# Neuropsychiatric Lupus, the Blood Brain Barrier, and the TWEAK/Fn14 Pathway

**DOI:** 10.3389/fimmu.2013.00484

**Published:** 2013-12-25

**Authors:** Ariel D. Stock, Jing Wen, Chaim Putterman

**Affiliations:** ^1^Department of Microbiology and Immunology, Albert Einstein College of Medicine, Bronx, NY, USA; ^2^Division of Rheumatology, Albert Einstein College of Medicine, Bronx, NY, USA

**Keywords:** TWEAK, Fn14, blood brain barrier, neuropsychiatric lupus, MRL/lpr

## Abstract

Patients with systemic lupus erythematosus (SLE) can experience acute neurological events such as seizures, cerebrovascular accidents, and delirium, psychiatric conditions including depression, anxiety, and psychosis, as well as memory loss and general cognitive decline. Neuropsychiatric SLE (NPSLE) occurs in between 30 and 40% of SLE patients, can constitute the initial patient presentation, and may occur outside the greater context of an SLE flare. Current efforts to elucidate the mechanistic underpinnings of NPSLE are focused on several different and potentially complementary pathways, including thrombosis, brain autoreactive antibodies, and complement deposition. Furthermore, significant effort is dedicated to understanding the contribution of neuroinflammation induced by TNF, IL-1, IL-6, and IFN-γ. More recent studies have pointed to a possible role for the TNF family ligand TWEAK in the pathogenesis of neuropsychiatric disease in human lupus patients, and in a murine model of this disease. The blood brain barrier (BBB) consists of tight junctions between endothelial cells (ECs) and astrocytic projections which regulate paracellular and transcellular flow into the central nervous system (CNS), respectively. Given the privileged environment of the CNS, an important question is whether and how the integrity of the BBB is compromised in NPSLE, and its potential pathogenic role. Evidence of BBB violation in NPSLE includes changes in the albumin quotient (*Q*_alb_) between plasma and cerebrospinal fluid, activation of brain ECs, and magnetic resonance imaging. This review summarizes the evidence implicating BBB damage as an important component in NPSLE development, occurring via damage to barrier integrity by environmental triggers such as infection and stress; cerebrovascular ischemia as result of a generally prothrombotic state; and immune mediated EC activation, mediated by antibodies and/or inflammatory cytokines. Additionally, new evidence supporting the role of TWEAK/Fn14 signaling in compromising the integrity of the BBB in lupus will be presented.

## Introduction

Systemic lupus erythematosus (SLE) is a systemic autoimmune disease characterized by hyper-activation of B and T lymphocytes resulting in the overproduction of autoantibodies, tissue deposition of immune complexes, and high levels of inflammatory cytokines, cumulatively resulting in a systemic pro-inflammatory state (1). SLE patients may suffer from skin, joint, hematologic, and renal disease, the latter being a predominant contributor to morbidity and mortality. Treatments have traditionally consisted of corticosteroids and potent immunosuppressive agents such as cyclophosphamide, though biologic medications targeting particular cytokines may eventually prove to be promising alternatives. Additionally, the course of the disease is highly variable between patients, with certain manifestations more common than others, and the overall impact on quality of life dependent on the individual patient’s circumstances and particular disease manifestations.

Central nervous system (CNS) presentations in SLE patients consist of a broad array of symptoms, which can be generally divided between focal neurological and diffuse psychiatric manifestations. Focal episodes may include seizures and cerebrovascular events, while psychiatric presentations often consist of anxiety and depression (2). A neuropsychiatric SLE (NPSLE) phenotype can be a presenting feature of lupus, and is eventually found in up to 40% of SLE patients (3). Research into the underlying mechanisms of NPSLE has taken several different and potentially complementary directions. Human, murine and *in vitro* systems have all been utilized to examine the effects of autoantibodies, cytokines, vascular disease, and cellular effectors in the development of NPSLE symptoms. TNF-like weak inducer of apoptosis, better known as TWEAK, is a TNF family member cytokine which together with its sole confirmed receptor Fn14 have recently shown to be instrumental in the pathogenesis of murine NPSLE. Furthermore, it is increasingly evident that blood brain barrier (BBB) disruption is an essential component of NPSLE pathogenesis (4), and that TWEAK may play an important role in this process (5).

## NPSLE in Human Lupus and Experimental Models

Studying NPSLE in humans poses some obvious limitations, including the scarcity of CNS tissue samples and the heterogeneity of NPSLE presentations. Most available data from NPSLE patients consist of blood and cerebrospinal fluid (CSF) analysis, and radiologic imaging, including magnetic resonance imaging (MRI). CSF is often remarkable for the presence of increased immunoglobulins, elevated concentrations of cytokines, and evidence of BBB disruption, as measured by increased albumin concentrations. MRI data has additionally proven useful in identifying the brain regions most frequently involved in NPSLE, as well as which CNS tissues are affected (6). Additionally, extensive work has gone into the correlation between certain systemic autoantibody titers and NPSLE phenotypes (7).

There are several spontaneous mouse models of SLE, including the NZB × NZW F1 (NZB/W F1), BXSB, and MRL/*Tnfrsf6^lpr/lpr^* (MRL/lpr) strains. These three models all develop some measure of neuropsychiatric disease. BXSB mice, for example, demonstrate problems with both spatial and non-spatial learning tasks (8). One issue with the BXSB model, however, is the sex bias toward males, which is inconsistent with the strong female predominance found in human SLE. Additionally, BXSB and NZB/W F1 mice may have congenital structural abnormalities of the brain (9), potentially confounding structure-function analyses. Both NZB/W F1 and MRL/lpr mice demonstrate neurological deficits (10, 11), though the MRL/lpr model has a greater incidence of neuropsychiatric disease (12). The MRL/lpr has the added benefit of a congenic control (MRL^+/+^), which does not develop disease.

The MRL/lpr strain develops a disease phenotype consistent with the affective and behavioral pathologies seen in human lupus (13). Gao et al. found that depressive symptoms appear as early as 6 weeks of age in female MRL/lpr mice, preceding onset of renal pathology. Additionally, we found a correlation between depressive symptoms and several autoantibodies, including anti-NMDAR and anti-dsDNA (14). Similarly, depression and other neuropsychiatric symptoms can appear early in the disease course in human disease (15). MRL/lpr mice demonstrate increased immobility on the forced swim test, which is a widely accepted indicator of depression in rodents (if strength and locomotion are otherwise normal). Additionally, MRL/lpr mice display decreased preference for sweetened water (anhedonia), as well as an acquired anosmia, both manifestations of murine depressive-like behavior (16, 17). Finally, cognitive tests in MRL/lpr mice reveal clear deficits in the object placement task indicating deficits in spatial memory, relatable to the cognitive decline found in NPSLE patients.

Another experimental model of NPSLE is induced by treatment with anti-*N*-methyl-d-aspartate (anti-NMDA; also known as anti-NR2) receptor antibodies coupled with BBB disruption in BALB/c mice (18). Depending on the method of BBB disruption, resulting symptoms may include impaired performance on memory tasks or altered fear responses (19, 20). More recently, Kivity et al. showed that intracerebroventricular transfer of the 16/6 idotype (a human anti-ssDNA antibody) into C3H mice resulted in hippocampal inflammation and decreased performance in memory tasks (21).

## Neuropathic Antibodies in NPSLE

As previously mentioned, NPSLE deficits can be defined as either focal or diffuse in nature. Focal findings in NPSLE are most readily associated with the presence of antiphospholipid (aPL) antibodies, including anti-cardiolipin antibodies, anti-β2glycoprotein I antibodies, and lupus anticoagulant (22). These antibodies dramatically increase susceptibility to thrombosis, resulting in an increased rate of cerebrovascular accidents (CVA) and transient ischemic attacks (TIA). It is thought that aPL antibodies may act through increasing oxidative stress, as measured by increased level of oxidized low-density lipoprotein, which itself is associated with atherosclerosis and thrombosis (23). While these patients will present with typical focal findings, such as motor and cranial nerve deficits, seropositivity for aPL antibodies is not typically associated with diffuse psychiatric and cognitive presentations (7).

Other circulating and intrathecal antibodies are also associated with NPSLE manifestations. Anti-ribosomal-P (anti-P) antibodies have long been associated with NPSLE presentations (24–26), and more recently have been found to induce depression in mice when injected intraventricularly (16). Recent work by Matus et al. found that anti-P antibodies from human lupus serum induced calcium influx and subsequent apoptosis in p331 positive neurons in rats, which they characterized as a new P-antigen. Death of these neurons, found in the hippocampus, amygdala, and certain neocortical layers, account for a broad range of potential symptoms, including depression, memory deficits, and cognitive decline (27). Anti-NMDA receptor antibodies are also associated with psychiatric symptoms, such as depression and memory dysfunction in SLE patients, and altered fear responses when transferred to mice, due to excitotoxic glutamatergic effects on neurons (14, 19, 28–30). Anti-U1-RNP antibodies have been reported by Sato et al. as a more specific marker of NPSLE than anti-P or anti-NR2 antibodies (31), and may potently induce expression of interferons (32). Anti-ganglioside (anti-GM1) antibodies were once thought to be associated with NPSLE pathogenesis (4, 33) through disruption of voltage gated Na^+^ channels found near nodes of Ranvier (34), though it has since been found that they are more likely associated with peripheral neuropathies than central NPSLE presentations (35). Other antibodies associated with NPSLE include anti-dsDNA, anti-Microtubule-associated Protein 2, anti-Triose-phosphate isomerase, and brain reactive autoantibodies (BRAA) (36–38). The reader is referred to a number of excellent reviews for further detail regarding these and other neuropathic antibodies (39–42). Regardless of the antigenic specificity of these neuropathic autoantibodies, the question remains how they gain entry into the central nervous system (CNS) from the systemic circulation, implicating a failure in the BBB as a key component of NPSLE pathogenesis.

## The Blood Brain Barrier

The CNS is maintained as a privileged environment due to the combination of tight junctions between endothelial cells (ECs) limiting paracellular transport, and astrocytic processes, which regulate transcellular transport from systemic circulation. The choroid plexus (CP) and arachnoid epithelia similarly maintain this barrier through tight junctions, as well as secrete and reabsorb CSF, respectively. Another element that seems essential to a competent blood brain barrier is the presence of resident microglia. Microglia populations of monocytic origin are found perivascularly within the CNS parenchyma, limit paracellular transport across ECs, and may serve as a bridge between CNS and systemic immune activity (43).

The isolating nature of brain microvascular ECs is attributable to the tight junctions, including members of the zonula occludens (ZO-1) and claudin (claudin-5) families, resulting in impermeability to macromolecules (44). Additionally, these multi-laminated junctional complexes provide very high electrical resistance, dependent on the basal parenchymal presence of astrocytes, yielding remarkably low permeability to smaller and ionic molecules (45, 46). Astrocytes are also required to provide the environmental cues ECs need to develop and maintain their unique CNS phenotype, and are involved in bidirectional cytokine signaling with ECs (47). Microglia are involved as well in modulating EC through secretion of TNF, which upregulates MHC-I presentation on ECs and promotes entry of T-cells into brain parenchyma during pathological states (48).

Beyond serving as a barrier, ECs are directly responsible for regulating the immune response in the brain. ECs help maintain the CNS in its basal state of suppressed immunity through secretion of TGFβ (49) and soluble cellular adhesion molecules (50). Brain ECs can, however, activate an immune response, such as under LPS stimulation, which induces production of IL-6 and GM-CSF. Interestingly, Verma et al. showed that LPS itself does not appear to induce disruption of EC junctional complexes; rather, further signaling by luminal and parenchymal effector cells likely potentiates BBB disruption following LPS stimulation (51).

The CP vasculature is unique within the CNS, as it consists of fenestrated ECs necessary for CSF volume maintenance. This vasculature is isolated from brain parenchyma by a blood-CSF barrier (BCSFB). The BCSFB consists of cuboidal CP epithelial cells, which are interconnected by tight junctions, effectively providing for a barrier similar to the BBB (52, 53). CSF not only provides physical support to CNS tissue by reducing its apparent weight, it is also able to rapidly transmit hormonal signals within the CNS (54) and provide for drainage, much as the lymphatics do systemically (55). Additionally, CSF is enriched with memory T-cells due to one of its primary roles, immune surveillance of the CNS. Normally, non-autoreactive T-cells are allowed to pass through the CP epithelial barrier unhindered (56). It is believed, however, that through increased expression of cellular adhesion molecules, CP ECs are involved in the pathogenesis of certain autoimmune conditions, such as multiple sclerosis (57).

## Evidence of BBB Damage in SLE

There are several markers available to monitor the integrity of the BBB. Albumin is a large and extensively charged protein that is not synthesized intrathecally, and whose transport into the CNS is tightly regulated. As such, an elevated albumin concentration gradient (*Q*_alb_) between CSF and plasma (normal [Alb]_CSF_/[Alb]_Plasma_ ≤ 7.6 × 10^−3^) serves as an indicator of BBB disruption, and has been found repeatedly in NPSLE patients (31, 58, 59) and MRL/lpr mice (60). While *Q*_alb_ provides a useful measurement of relatively large scale leakage across the BBB, it lacks the finer resolution needed to appreciate small and transient leakage (4). The IgG index [CSF_(IgG/Albumin)_]/[Serum(_IgG/Albumin)_] is another useful measure of BBB permeability that can also identify the relative intrathecal vs. systemic origin of IgG within the CNS (61, 62), and is found elevated in both NPSLE patients and experimental models (31, 60). Sato et al. found both an elevated *Q*_alb_ and an elevated IgG index in 8 and 9 of 14 NPSLE patients, respectively (31). Similarly, Sidor et al. found elevated *Q*_alb_ and IgG index measurements in MRL/lpr mice when compared with MRL^+/+^ mice, along with increased neurodegeneration in those mice with a disrupted BBB (60). Jacob et al. further demonstrated that IgG enters CNS parenchyma in MRL/lpr mice (63). Finally, Ma et al. have shown extensive penetration of CD3^+^ cells into the CP and in brain parenchyma, as well as the presence of CD19^+^-B-cells in MRL/lpr mice, providing further evidence of BBB failure in NPSLE (64).

The availability of new and improved modalities with finer resolution will likely continue to be an asset in measuring BBB function non-invasively in NPSLE. Since the calcium binding protein S100B is predominately found in astrocytes, its presence in serum serves as valuable indicator of BBB injury (65–67). Schenatto et al., examining a cohort of 89 SLE patients, identified elevated S100B levels in NPSLE vs. non-NPSLE, a finding which was even more prominent during acute episodes (68). Evolution of MRI is also proving to be useful in the clinical characterization of BBB breaches. The increasing availability of 3.0 T MRI magnets and newer gadolinium (Gd) containing contrast agents, which visualizes contrast flow into CNS parenchyma with T1-weighted imaging, are improving the ability to highlight areas of BBB insufficiency (69). Recently, Toledano et al. utilized multiple MRI modalities in imaging NPSLE patient brains, and found vascular damage in a third of patients with the vast majority consisting of small vessel damage (70). One could reasonably speculate that evidence of BBB disruption may be obscured due to transient changes in BBB integrity, or the reparative effects of treatment. Indeed, findings of BBB disruption by Gd-contrast enhancement are likely under-reported, since the frequent use of corticosteroids in SLE treatment likely results in stabilization of BBB damage (71).

## Mechanisms of Brain EC Activation and BBB Disruption in SLE

### EC activation in SLE

As described above, brain ECs are not mere bystanders in the regulation of the CNS environment; they play an active role in concert with both CNS and luminal effector cells and molecules. Infection and systemic inflammation are potent activators of ECs, resulting in upregulated expression of cytokines and chemokines, thereby potentiating a local immune response (51, 72). IL-1, TNF, and LPS signaling each result in upregulation of E-selectin, ICAM-1, and VCAM-1 in microvascular ECs *in vitro* (73). MRL/lpr mice are found to have elevated expression of ICAM-1 and VCAM-1 in predominately CP associated ECs when compared to congenic controls (74). Sun et al. have recently shown that immune complexes in SLE induce production of inflammatory cytokines and cellular adhesion molecules in ECs via NF-κB signaling, due to HMGB1-RAGE axis activation (75).

TREX1 is a major endogenous 3′–5′ DNA exonuclease. Mutations in TREX1 are associated with chilblain lupus erythematosus, a rare form of cutaneous disease, as well as with sporadic SLE (76). In addition, TREX1 variants are found in two other diseases with neurological manifestations, autosomal dominant retinal vasculopathy with cerebral leukodystrophy and Aicardi–Goutieres syndrome (77). TREX1 deficiency or dysfunction may lead to accumulation of cytosolic DNA and enhanced alpha-interferon signaling. Furthermore, TREX1 deficiency in lymphocytes modulates vascular EC angiogenesis (78), suggesting an interesting possible link between the genetic susceptibility for lupus associated with TREX1, ECs, and CNS disease (76). Irrespective of the cause, endothelial activation results in increased vascular permeability and diapedesis, and increased local aggregation of humoral and cellular effectors.

### Environmentally induced BBB disruption

Diamond et al. have done extensive work on anti-NMDA receptor antibodies as effectors of NPSLE symptomatology. In their early studies, CNS disease was induced by direct injection of anti-NMDA receptor antibodies into the cerebral cortices of healthy mice, thereby bypassing the BBB (79). They then utilized several models of extraneous BBB disruption, including LPS as model of infection and epinephrine as a model of stress. When LPS was used to induce BBB disruption, mice showed poorer performance on the Morris water maze and T-maze tasks, indicative of learning and memory deficits, and consistent with hippocampal damage. Neuron loss in the hippocampus was found histologically and was evident as structural abnormalities on MRI (80). When using epinephrine, neuron loss occurred selectively in the amygdala, inducing alterations in conditioned fear responses in mice, while sparing hippocampal neurons (81). These findings provide a possible explanation for the phenotypic differences between NPSLE patients, and the role environmental mediators may play.

### Anti-endothelial cell antibodies

Conti et al. found that 64.7% of NPSLE patients sera are positive for anti-endothelial cell antibodies (AECAs), as compared to only 29.4% of non-NPSLE patients (82). AECAs have been previously characterized as inducers of increased EC cellular adhesion molecules, including E-selectin, ICAM-1, and VCAM-1. Nara et al. stimulated HUVEC with monoclonal antibodies targeted toward thrombomodulin, a proposed antigenic substrate of some AECAs in SLE patients (83), and found increased endothelial production of IL-6 and IL-8 mediated through the NF-κB pathway (84). More recently, Yoshio et al. found that anti-NR2 antibodies recognize antigenic targets on HUVECs, and can induce IL-6 and IL-8 expression with IL-1β co-treatment (85). Collectively, AECAs may induce endothelial activation, which is pivotal in many inflammatory processes, but in the brain is an important component of BBB disruption as well (86).

### Complement

One of the hallmarks of increased disease activity in SLE patients is the depletion of complement components from serum, due to consumption by circulating immune complexes, deposition in target tissues and targeting by anti-C1q antibodies (87, 88). Low circulating levels of complement components C3 and C4 have also been suggested as potential biomarkers of human NPSLE activity (89). Alexander et al. first demonstrated that complement inhibition can attenuate NPSLE presentations in MRL/lpr mice (90). Jacob et al. then showed that C5aR activation induces EC cytoskeletal alteration *in vitro* and laminin disruption in MRL/lpr brain vasculature, both indicators of BBB damage. Additionally, C5aR activation results in increased CCL2 and CXCL2 production by mouse microvascular ECs, when pretreated with IL-6 (91). Finally, in further characterizing the BBB disruption that occurs with C5aR activation, Jacob et al. found increased expression of MAP-kinase, increased nuclear NF-κB translocation and decreased zona occludin (ZO) levels, indicative of EC junctional complex interruption, which would dramatically increase BBB permeability (92).

### Cytokines and chemokines

Stimulation of human brain microvessel ECs (HBMEC) with cytokines such as IL-1β, IL-8, TNF, and IFN-γ are known to induce increased permeability across monolayers (86, 93). CCL2 signaling has been shown to play a role in BBB disruption both *in vitro* and in CCR2^−/−^ mice (94, 95). Zameer et al. found elevated expression levels of ICAM-1 and VCAM-1 in the CNS of MRL/lpr mice, providing further evidence of an inflammatory process involving the BBB (74, 96). Trysberg et al., analyzing CSF from lupus patients, found elevated levels of IL-6 and IL-8 which were correlated with elevated MMP-9 levels, the latter associated with degradation of BBB extracellular matrix (97).

In most, if not all of the above models of BBB disruption, it is clear that the cytokine and chemokine environment is critical. While complement and AECAs may indeed be effectors of BBB disruption, they cannot do so on their own; cytokines are needed to fully activate and disrupt the BBB. Amongst these cytokines, TWEAK has recently been demonstrated as a potent effector of multiple downstream pathways needed for BBB disruption, including activating intracellular signaling cascades and inducing production of additional cytokines, chemokines, and metalloproteinases.

The causes and consequences of EC activation are summarized in Table 1.

**Table 1:** **Potential routes of endothelial cell activation in SLE**.

Cause of endothelial activation	Model	Findings
Environmental mediators	BBB disruption by treatment with LPS or epinephrine, in models of infection and stress, respectively (19, 81)	Treatment with human lupus serum containing anti-NMDAR antibodies following BBB disruption results in IgG deposition, hippocampal neuron loss, and memory impairment (LPS), or amygdala neuron loss and altered fear responses (epinephrine)
AECAs	*In vitro* treatment of HUVEC with anti- thrombomodulin (83, 84) or anti-NR2 antibodies (85)	Increased IL-6 and IL-8 expression
Complement	Mouse brain endothelial cells and MRL/lpr mice (63, 92)	C5aR activation yields increased CCL2 and CXCL2, NFκB signaling, and decreased ZO expression
Cytokine and chemokines	MRL/lpr mice (74) and CSF from human lupus patients (97)	Elevated ICAM-1 and VCAM-1 in MRL/lpr CNS; increased IL-6, IL-8, and MMP-9 in lupus CSF
TWEAK	*In vitro* hCMEC/D3 cells (5)	Elevated ICAM-1, CCL2, IL-6, IL-8, and MMP-9, ZO-1 degradation and decreased occludin levels

### TWEAK, NPSLE, and BBB disruption

TWEAK is a pro-inflammatory cytokine member of the TNF superfamily. Through activation of its sole receptor, Fn14, TWEAK variably induces cellular proliferation, angiogenesis, inflammation, and apoptosis (98). As previously mentioned, MRL/lpr mice develop a neuropsychiatric phenotype remarkably similar to human SLE. We recently found increased TWEAK and Fn14 expression in the cerebral cortices of MRL/lpr mice. Furthermore, in MRL/lpr Fn14-knockout (Fn14KO) mice we found significantly improved cognitive function, decreased depression, and less anhedonia, as demonstrated by object placement tasks, the forced swim test, and preference for sweetened water, respectively (17). The attenuated neuropsychiatric phenotype in Fn14 deficient lupus mice may be due to decreases in brain expression of CCL5 and C3, which have been found associated with depression and cognitive decline (99–101). Other mechanisms by which the Fn14KO is protective of NPSLE development in MRL/lpr mice are under active investigation.

**Figure 1:**
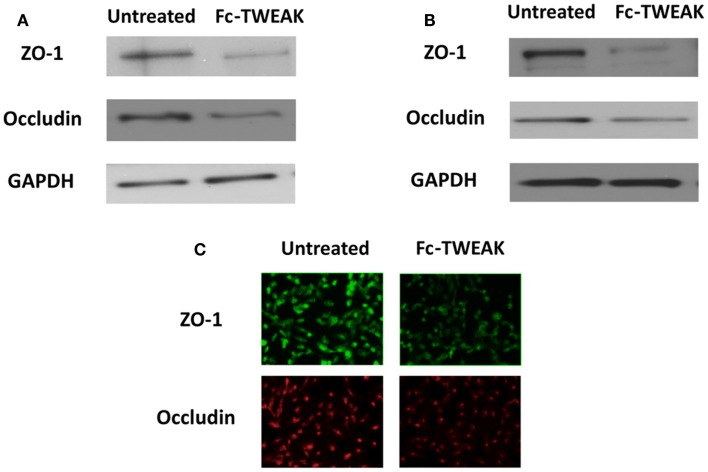
**ZO-1 and occludin expression after treatment with Fc-TWEAK**. ZO-1 and occludin expression was measured by western blot. Treatment with Fc-TWEAK (100 ng/mL, 48 h) decreased ZO-1 and occludin expression in hCMEC/D3 cells **(A)**. ZO-1 and occludin expression was decreased in hBMEC cells after treatment with Fc-TWEAK (100 ng/mL, 24 h) as well **(B)**. Similarly, immunofluorescent detection of ZO-1 and occludin in hBMECs was reduced after treatment with Fc-TWEAK (100 ng/mL, 24 h) **(C)**.

Fn14 is expressed both in the human cerebral microvascular EC line (hCMEC/D3) and astrocytes, while TWEAK is secreted by only the latter (5, 102). As described above, we found decreased *Q*_alb_ ratios in MRL/lpr Fn14KO mice as well as decreased CSF titers of anti-dsDNA antibodies (17). More recently, we characterized the effects of TWEAK on endothelial cytokine expression and barrier disruption using hCMEC/D3 brain ECs, and found TWEAK-induced increases in ICAM-1, CCL2, IL-6, and IL-8 (5). Furthermore, TWEAK-induced activation of the MAPK pathway yielded increased expression of MMP-9, which degraded ZO-1, decreased occludin expression (Figure 1), and increased permeability (5). A similar finding was seen by Polavarapu et al. in their investigation of cerebral ischemic injury, where intracerebral injection of TWEAK in wild type mice increased MMP-9 activity and BBB permeability (103). Furthermore, TWEAK induced expression of ICAM-1, IL-8, and IL-6 in cultured astrocytes, typical of reactive astrocytes, another effector of BBB disruption (104). Together, this data supports the conclusion that TWEAK is instrumental in the development of lupus associated neuropsychiatric disease, with BBB disruption as an important mechanistic contribution of this cytokine. The lack of effect of TWEAK on systemic autoantibodies (17) and our current understanding of the mechanism of action of the TWEAK/Fn14 signaling pathway also suggest that attenuated neuropsychiatric disease in MRL/lpr Fn14 KO mice is not due to a reduced systemic autoantibody response, but rather local (i.e., brain) effects of blocking this pathway such as the effect on the BBB discussed here. Finally, it is important to note that not only is TWEAK/Fn14 signaling involved in NPSLE, but this cytokine/receptor pair has been implicated in the pathogenesis of injury in other major lupus target organs, including the kidney [for which there is evidence both in murine models (105, 106), and in human disease (107)], and in cutaneous lupus as well (108).

The success of mAb treatment targeting pathogenic cytokines such as TNF, IL-1, and BLyS in inflammatory rheumatic diseases, together with the data presented above, strongly suggest the need to examine anti-TWEAK antibody treatment as novel treatment approach in NPSLE. Antibodies given intravenously can have therapeutic effects on the brain, especially if the blood brain barrier is already breached (as in NPSLE) (109). Alternatively, it would be necessary to bypass the BBB, or develop a delivery system to deliver the antibody to the CNS despite the compartmentalization of the brain from the blood (e.g., bispecific antibodies using the transferrin receptor) (110).

## Conclusion

Neuropsychiatric SLE is often associated with the presence of neuropathic antibodies within the CNS, making the question of how they gain entry into this anatomically privileged space increasingly important. Evidence points to entry of autoantibodies across the BBB, with entry into different brain regions and specific autoantibody subtypes potentially associated with the variable phenotypes found in both murine experimental models and NPSLE patients. There is strong support for the roles of AECAs, complement components, and environmental mediators in increasing permeability across the BBB, though in each of these cases, cytokines and chemokines have an essential role as well. TWEAK appears to be one such cytokine that is necessary for the development of NPSLE; one mechanism central to the contributions of the TWEAK/Fn14 axis appears to be its role in BBB disruption.

Interestingly, TWEAK/Fn14 signaling has been implicated in other neurologic diseases besides NPSLE, including hypoxic brain damage and autoimmune brain disease. Elsewhere in this special issue, Yepes describes how TWEAK/Fn14 signaling in middle cerebral artery occlusion (a model of ischemic stroke) induces inflammation and MMP-9 mediated basal lamina disruption (111). Desplat-Jego et al. demonstrated the involvement of TWEAK in the development of experimental autoimmune encephalomyelitis (EAE), a murine model of multiple sclerosis, and that TWEAK over-expression in transgenic mice further exacerbates the EAE phenotype (102). In both these disease models, preventing TWEAK signaling via Fn14 deficiency, treatment with a Fn14-Fc decoy receptor, or treatment with anti-TWEAK monoclonal antibodies results in attenuated disease. Ameliorating the disruption of the BBB may be a valuable tool in the control of NPSLE as well other neurologic disorders, and targeting the TWEAK pathway may be one way to do so.

## Conflict of Interest Statement

The authors Ariel D. Stock and Jing Wen declare that the research was conducted in the absence of any commercial or financial relationships that could be construed as a potential conflict of interest. Research presented in this paper was supported in part by a grant from Biogen Idec to Chaim Putterman.
